# Supplementary material for: Compressed sensing expands the multiplexity of imaging mass cytometry
Source: Nat Commun. 2025 Nov 27;16:11671. doi: 10.1038/s41467-025-66629-4 (PMC12748953; doi:10.1038/s41467-025-66629-4)
Supplement: Supplementary file 4 — Reporting Summary [file 41467_2025_66629_MOESM4_ESM.pdf]

nature portfolio

Corresponding author(s): Bernd Bodenmiller

Last updated by author(s): Nov 6, 2025

Reporting Summary

Nature Portfolio wishes to improve the reproducibility of the work that we publish. This form provides structure for consistency and transparency in reporting. For further information on Nature Portfolio policies, see our Editorial Policies and the Editorial Policy Checklist.

Statistics

For all statistical analyses, confirm that the following items are present in the figure legend, table legend, main text, or Methods section.

n/a	Confirmed	
☐	☒	The exact sample size (*n*) for each experimental group/condition, given as a discrete number and unit of measurement
☐	☒	A statement on whether measurements were taken from distinct samples or whether the same sample was measured repeatedly
☒	☐	The statistical test(s) used AND whether they are one- or two-sided<br>*Only common tests should be described solely by name; describe more complex techniques in the Methods section.*
☒	☐	A description of all covariates tested
☒	☐	A description of any assumptions or corrections, such as tests of normality and adjustment for multiple comparisons
☐	☒	A full description of the statistical parameters including central tendency (e.g. means) or other basic estimates (e.g. regression coefficient) AND variation (e.g. standard deviation) or associated estimates of uncertainty (e.g. confidence intervals)
☒	☐	For null hypothesis testing, the test statistic (e.g. *F*, *t*, *r*) with confidence intervals, effect sizes, degrees of freedom and *P* value noted<br>*Give P values as exact values whenever suitable.*
☒	☐	For Bayesian analysis, information on the choice of priors and Markov chain Monte Carlo settings
☒	☐	For hierarchical and complex designs, identification of the appropriate level for tests and full reporting of outcomes
☐	☒	Estimates of effect sizes (e.g. Cohen's *d*, Pearson's *r*), indicating how they were calculated

Our web collection on statistics for biologists contains articles on many of the points above.

Software and code

Policy information about availability of computer code

Data collection	Raw IMC data was acquired using Standard Biotool CyTOF imaging mass cytometry software (v7.0).
Data analysis	Steinbock v0.16.0 (https://github.com/BodenmillerGroup/steinbock) was used to convert the ion count raw data obtained from IMC software into single-cell expression data.<br>All code used for preprocessing single-cell expression data, SMAF dictionary calculation, and decompressing composite data are available at https://github.com/BodenmillerGroup/CISI-IMC.<br>R: 4.2.0 (IMC preprocessing and plotting figures)<br>Python: 3.9 (CISI dictionary calculation and decompression)

For manuscripts utilizing custom algorithms or software that are central to the research but not yet described in published literature, software must be made available to editors and reviewers. We strongly encourage code deposition in a community repository (e.g. GitHub). See the Nature Portfolio guidelines for submitting code & software for further information.

Data

Policy information about availability of data

All manuscripts must include a data availability statement. This statement should provide the following information, where applicable:

- Accession codes, unique identifiers, or web links for publicly available datasets
- A description of any restrictions on data availability
- For clinical datasets or third party data, please ensure that the statement adheres to our policy

Tiff files for IMC data and single-cell data generated in this study have been deposited in Zenodo (DOI: 10.5281/zenodo.17533580)

Research involving human participants, their data, or biological material

Policy information about studies with human participants or human data. See also policy information about sex, gender (identity/presentation), and sexual orientation and race, ethnicity and racism.

Reporting on sex and gender	Sex and gender were not considered, since this work is not biological study but rather demonstrating a method.
Reporting on race, ethnicity, or other socially relevant groupings	Race,ethnicity, or other socially relevant groupings were not considered, since this work is not biological study but rather demonstrating a method.
Population characteristics	Tissue samples were from patients who donated to the University Hospital Zurich. We did not analyze population characteristics as this work is not a biological study, but is demonstrating a method.
Recruitment	Subjects were not recruited for this study. Tissue samples were from patients who donated to the University Hospital Zurich.
Ethics oversight	Use of samples received from University Hospital Zurich was approved by the Ethikkommission Kanton Zürich (KEK-ZH-Nr. 2014-0604, 100TO).

Note that full information on the approval of the study protocol must also be provided in the manuscript.

Field-specific reporting

Please select the one below that is the best fit for your research. If you are not sure, read the appropriate sections before making your selection.

[x] Life sciences   [ ] Behavioural & social sciences   [ ] Ecological, evolutionary & environmental sciences

For a reference copy of the document with all sections, see nature.com/documents/nr-reporting-summary-flat.pdf

Life sciences study design

All studies must disclose on these points even when the disclosure is negative.

Sample size	We used 71 tissues for validation dataset. Sample size was not calculated since this work is not biological study but rather demonstrating a method. CISI-IMC was validated on multiple tissues and therefore successfully supported the versatility of the method.
Data exclusions	Region without significant portion of immune or stromal cells were excluded for the training dataset.
Replication	For the dictionary calculation, 10 replication was performed to assess the reproducibility, and all attempts were successful.
Randomization	Randomization is not relevant as this work is not a biological study. However, regions to image in tissue slide were selected randomly. Both of training dataset and validation dataset were selected to cover as many type of tissue and immune/stromal cell types.
Blinding	Blinding is not relevant as this work is not a biological study.

Reporting for specific materials, systems and methods

We require information from authors about some types of materials, experimental systems and methods used in many studies. Here, indicate whether each material, system or method listed is relevant to your study. If you are not sure if a list item applies to your research, read the appropriate section before selecting a response.

Materials & experimental systems

n/a	Involved in the study
☐	☒ Antibodies
☒	☐ Eukaryotic cell lines
☒	☐ Palaeontology and archaeology
☒	☐ Animals and other organisms
☒	☐ Clinical data
☒	☐ Dual use research of concern
☒	☐ Plants

Methods

n/a	Involved in the study
☒	☐ ChIP-seq
☒	☐ Flow cytometry
☒	☐ MRI-based neuroimaging

Antibodies

Antibodies used
The list of antibody and their suppliers, clone names, concentrations are available in Supplementary Data 1

Validation
All antibodies were validated by testing on several tissues such as tonsil. Expected spatial distribution and expression profile was confirmed for all antibodies.

Plants

Seed stocks
Report on the source of all seed stocks or other plant material used. If applicable, state the seed stock centre and catalogue number. If plant specimens were collected from the field, describe the collection location, date and sampling procedures.

Novel plant genotypes
Describe the methods by which all novel plant genotypes were produced. This includes those generated by transgenic approaches, gene editing, chemical/radiation-based mutagenesis and hybridization. For transgenic lines, describe the transformation method, the number of independent lines analyzed and the generation upon which experiments were performed. For gene-edited lines, describe the editor used, the endogenous sequence targeted for editing, the targeting guide RNA sequence (if applicable) and how the editor was applied.

Authentication
Describe any authentication procedures for each seed stock used or novel genotype generated. Describe any experiments used to assess the effect of a mutation and, where applicable, how potential secondary effects (e.g. second site T-DNA insertions, mosiacism, off-target gene editing) were examined.
